# Supplementary material for: Transcriptomic Insights into Mechanisms of Early Seed Maturation in the Garden Pea (Pisum sativum L.)
Source: Cells. 2020 Mar 23;9(3):779. doi: 10.3390/cells9030779 (PMC7140803; doi:10.3390/cells9030779)

Sprint−2(10) vs Zhewa

Global Summary

%DE = 0.9
genes with fdr < 0.2 = 533 (264 + / 269 −)
genes with fdr < 0.1 = 465 (237 + / 228 −)
genes with fdr < 0.05 = 422 (215 + / 207 −)
genes with fdr < 0.01 = 317 (153 + / 164 −)

genes in genesets = 0

<FC> = 0
<t−score> = 513.93
<p−value> = 0
<fdr> = 0.1

Portrait

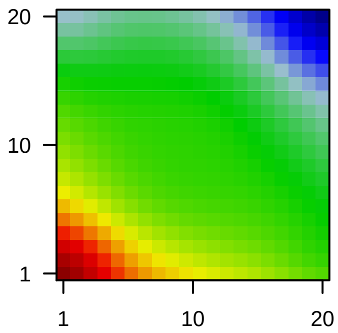

Top 100 DE genes

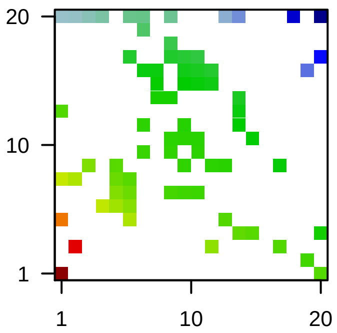

Global Genelist

Rank	ID	log(FC)	p−value	fdr	Metagene	Description
1	TRINITY_DN6	1.42	0e+00	4e−13	1 x 1	
2	TRINITY_DN1	−11.1	7e−15	9e−08	20 x 20	
3	TRINITY_DN1	−3.76	1e−09	4e−07	20 x 1	
4	TRINITY_DN1	2.31	1e−08	4e−07	1 x 1	
5	TRINITY_DN1	−0.53	1e−08	6e−07	4 x 20	
6	TRINITY_DN3	−18.89	4e−08	6e−07	1 x 20	
7	TRINITY_DN5	−0.19	4e−08	6e−07	7 x 19	
8	TRINITY_DN1	0	5e−08	3e−06	11 x 11	
9	TRINITY_DN3	−2.71	1e−07	3e−06	20 x 1	
10	TRINITY_DN4	5.32	2e−07	3e−06	1 x 1	
11	TRINITY_DN2	0	2e−07	3e−06	9 x 11	
12	TRINITY_DN8	−0.12	3e−07	3e−06	6 x 17	
13	TRINITY_DN4	−0.65	3e−07	3e−06	18 x 20	
14	TRINITY_DN5	−0.47	4e−07	3e−06	13 x 20	
15	TRINITY_DN3	−0.1	5e−07	3e−06	15 x 4	
16	TRINITY_DN6	−1.14	5e−07	5e−06	20 x 20	
17	TRINITY_DN4	0.07	6e−07	5e−06	6 x 7	
18	TRINITY_DN2	−0.05	6e−07	5e−06	11 x 15	
19	TRINITY_DN3	−4.05	7e−07	8e−06	20 x 20	
20	TRINITY_DN2	−0.1	1e−06	8e−06	10 x 17	

p−values

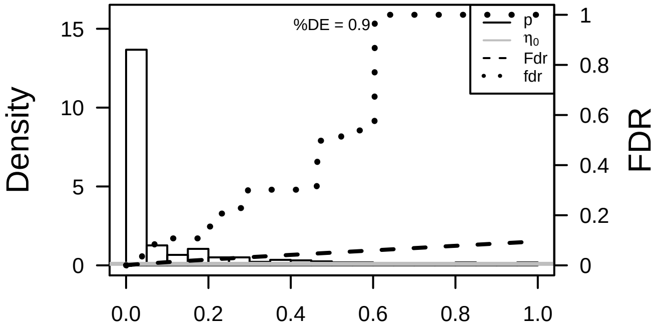

Supplement: Supplementary file 1 [file cells-09-00779-s001.zip › Supplementary materials/FileS3.zip!/FileS2/Hormones - Results/Summary Sheets - Differences/Reports/Sprint.2.10..vs.Zhewan.1.25..pdf]
